# Detection and characterization of small insertion and deletion genetic variants in modern layer chicken genomes

**DOI:** 10.1186/s12864-015-1711-1

**Published:** 2015-07-31

**Authors:** Clarissa Boschiero, Almas A. Gheyas, Hannah K. Ralph, Lel Eory, Bob Paton, Richard Kuo, Janet Fulton, Rudolf Preisinger, Pete Kaiser, David W. Burt

**Affiliations:** 1 The Roslin Institute and Royal (Dick) School of Veterinary Studies, University of Edinburgh, Easter Bush Campus, Midlothian, EH25 9RG UK; 2 Current Address: Departamento de Zootecnia, University of Sao Paulo/ESALQ, Piracicaba, SP 13418-900 Brazil; 3 Hy-Line International, Dallas Center, IA USA; 4 Lohmann Tierzucht GmbH, Cuxhaven, Germany

**Keywords:** Dindel, SAMtools, False discovery rate, InDel, Layer chicken, Loss-of-function mutation, Next generation sequencing

## Abstract

**Background:**

Small insertions and deletions (InDels) constitute the second most abundant class of genetic variants and have been found to be associated with many traits and diseases. The present study reports on the detection and characterisation of about 883 K high quality InDels from the whole-genome analysis of several modern layer chicken lines from diverse breeds.

**Results:**

To reduce the error rates seen in InDel detection, this study used the consensus set from two InDel-calling packages: SAMtools and Dindel, as well as stringent post-filtering criteria. By analysing sequence data from 163 chickens from 11 commercial and 5 experimental layer lines, this study detected about 883 K high quality consensus InDels with 93 % validation rate and an average density of 0.78 InDels/kb over the genome. Certain chromosomes, viz, GGAZ, 16, 22 and 25 showed very low densities of InDels whereas the highest rate was observed on GGA6. In spite of the higher recombination rates on microchromosomes, the InDel density on these chromosomes was generally lower relative to macrochromosomes possibly due to their higher gene density. About 43–87 % of the InDels were found to be fixed within each line. The majority of detected InDels (86 %) were 1–5 bases and about 63 % were non-repetitive in nature while the rest were tandem repeats of various motif types. Functional annotation identified 613 frameshift, 465 non-frameshift and 10 stop-gain/loss InDels. Apart from the frameshift and stopgain/loss InDels that are expected to affect the translation of protein sequences and their biological activity, 33 % of the non-frameshift were predicted as evolutionary intolerant with potential impact on protein functions. Moreover, about 2.5 % of the InDels coincided with the most-conserved elements previously mapped on the chicken genome and are likely to define functional elements. InDels potentially affecting protein function were found to be enriched for certain gene-classes e.g. those associated with cell proliferation, chromosome and Golgi organization, spermatogenesis, and muscle contraction.

**Conclusions:**

The large catalogue of InDels presented in this study along with their associated information such as functional annotation, estimated allele frequency, etc. are expected to serve as a rich resource for application in future research and breeding in the chicken.

**Electronic supplementary material:**

The online version of this article (doi:10.1186/s12864-015-1711-1) contains supplementary material, which is available to authorized users.

## Background

Small insertions and deletions (InDels) are the second most abundant kind of genetic variants in the genome after single nucleotide polymorphisms (SNPs) and are the most common type of structural variants (SV) [1, 2]. InDels have been implicated in many diseases and other traits. For instance, in human approximately one quarter of all known Mendelian diseases are associated with InDels [3]. In other organisms as well, such as in chicken, InDels have been found to be associated with different phenotypes e.g. growth [4], plumage colour [5], egg production [6], performance [7], body weight [8] and retinal degeneration and embryonic mortality [9]. Understanding InDels in greater detail is therefore important for profiling genetic variations within genomes [10], detecting causal mutations of genetic disorders [11, 12], studying the evolutionary relationship of species [13] and detecting footprints of selection [14, 15].

In spite of their importance and the massive advancements in high-throughput next-generation sequencing (NGS) technologies, the discovery of InDels has lagged behind that of SNPs for a number of reasons: (i) it is generally more difficult to map short reads by NGS covering InDel sites to the correct locations since this involves more complex gapped alignment or paired-end sequencing inference [16, 17]; (ii) since the read mappers align each fragment independently of other fragments, the InDels may appear as stretches of SNPs rather than as gaps [18]; (iii) the majority of InDels occur as short tandem repeats, which are difficult to map [19, 20]; (iv) higher coverage is required to detect InDels due to their relatively low frequency [2]; and (v) distinguishing true InDels from sequencing errors is difficult since there is no accurate sequencing error model for InDel [21]. InDel calling, therefore, requires a suitable genome aligner that can perform gapped alignment and software such as BWA [22, 23] and NOVOalign [23, 24] have been used for this purpose. Moreover, to reduce the problems of misalignments, local realignment of reads with InDels have been suggested and to minimise the detection of false positives due to other factors, stringent criteria for post-alignment filtrations needs to be applied [25].

Unlike SNPs, which are generated through point mutations, SVs (including InDels) can arise through a number of mechanisms, such as replication slippage, recombination, unequal crossing over and tandem duplications caused by imperfect repair of double-strand breaks [26–29]. As a consequence, InDel sizes vary widely from as small as 1 base to over several kilobases (kb) [10, 12, 30, 31]. This variation in size means that robust detection of SVs requires the use of a range of detection methods. Small SVs, like InDels, are frequently detected by mapping the small NGS sequencing reads against the reference genome. However, the size of the reads dictates the maximum size of InDels that can be detected by this method. In the 1000 Genomes Project Consortium [32], for example, this approach was used to detect InDels with a size range of 1–50 nucleotides.

In the present study we aimed to detect small InDels (less than 100 nucleotides) in the chicken genome, which is an important farm animal and a key model organism for genomic and developmental biology studies. Although a number of studies [14, 33–35] have focused on detecting SNPs from the chicken resulting in the discovery of millions of these variants, only a few studies [36–38] have analysed InDels. As a result the number of InDels reported in the public databases is quite low; for example dbSNP (build 140) reports only about 439 K InDels from the chicken genome. Only in a recent study, Yan *et al.* [38] have reported about 1.3 M InDels by analysing 12 diverse chicken lines. While this number has been a great contribution to the InDel database, study by Yan *et al.* analysed only single bird from each population and as a result could not shed light on certain aspects such as whether these InDels were segregating or fixed within populations, their allele frequencies, etc.

We used NGS sequence data generated from 163 chickens from 11 commercial and 5 experimental layer lines (samples pooled within lines) [34] to define our InDel set. To reduce the error rates often seen in InDel detection, we took only the consensus set called by two software packages, namely SAMtools [39] and Dindel [18], followed by stringent filtration steps. The approach of taking consensus variants from multiple callers has been used in many recent studies on different species [38, 40–42] . Although several InDel callers are now available, we used these two for a number of reasons. First, a recent study found that Dindel and SAMtools *mpileup* have the highest sensitivity in InDel calling at low coverage (less than 30X) compared to the other callers such as VarScan and GATK [16]. In our study the sequence coverage ranged between 7 and 17X for different populations and hence the choice of these packages appeared reasonable. Second, SAMtools is one of the most commonly used tools for detection of variants due to its simple workflow with many advanced features, such as its ability to perform local realignment [43]. Nevertheless, while the package has been reported to have a low false discovery rate (FDR) for SNP calling [44, 45], for InDel calling it was reported to have a rather high rate of 4.8 % on real data [18]. The Dindel package, on the other hand, has been modelled specifically for InDel calling and was found to have a much lower FDR (1.6 % on real data) compared to SAMtools [18, 16] but it has a complex workflow and long running time. Our study reports the discovery of about 883 K high quality consensus InDels using these two packages followed by stringent filtration criteria, and discusses the physical and functional characteristics of these InDels.

## Results

### InDel calling using SAMtools and Dindel

Both the SAMtools and Dindel packages use Bayesian models for the detection of InDels. SAMtools takes into account mapping qualities, base qualities and error rates (from raw sequence quality scores) in approximating the posterior probabilities of consensus genotypes, which are then used for calling the variants [17]. The SAMtools also performs local realignment around candidate InDel sites [43]. The basic principle underlying the Dindel package is to realign all reads mapped to a region to a number of candidate haplotypes of at least 120-bp length, which represent alternatives to the reference genome sequence [18]. The probabilistic realignment model of Dindel takes into account base qualities, map qualities of reads, insert size distribution and position-dependent rates of InDel error based on homopolymer run length. Upon realignment of the reads, InDels are called by comparing the posterior probabilities of pairs of haplotypes with and without InDels. Both tools provide Phred-based quality scores to the called variants that can be used for further filtration.

**Fig. 1:**
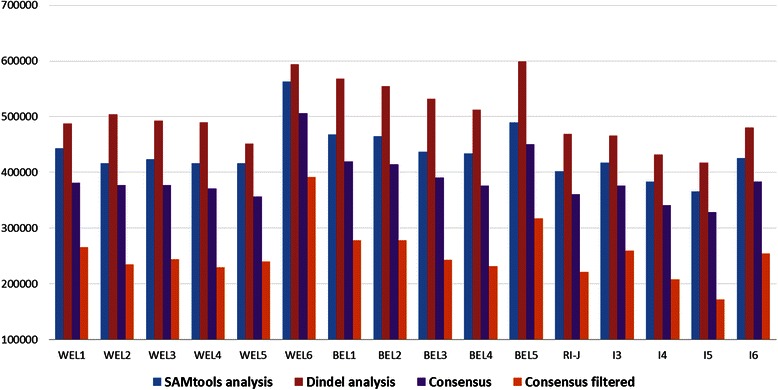
Number of InDels per line detected with SAMtools and Dindel packages. The consensus number of InDels was detected by both software and filtered. WEL = white egg layer; BEL = brown egg layer; I = inbred; RI-J = Roslin Institute. Chromosomes W and random are not included

The first stage of variant calling using SAMtools detected a total of 1,273,000 InDels from all the chicken lines. Only a few thresholds were incorporated within the commands for the initial calling of the variants, viz., minimum base quality of 20, minimum map quality of 20 and InDel alleles supported by at least two reads. More stringent filtration criteria were used later. In a parallel run, the Dindel package detected 6.4 % more InDels (n = 1,355,154) compared to SAMtools. The higher number of variants detected by Dindel was observed for each of the chicken lines analysed (Fig. 1, Additional file 1). One probable reason for calling a larger number of variants by Dindel is that unlike the SAMtools analysis, in Dindel, we did not set any minimum thresholds for base and map qualities as there were no options available to specify these parameters. The only initial criterion applied was the support of InDel alleles by at least two reads. Another possible reason could be that Dindel can use the information on insert size and the mate’s mapping quality for calling InDels in cases where one read of a pair failed to map to the correct location [18].

Even though the quality scores for individual variants were different from the two packages and the scores from SAMtools were 16–34 % higher compared to those from Dindel (the average quality scores were 108 and 83, respectively), there was a significant correlation between the scores generated by the two packages (r ≈ 0.36; *P* < 0.001).

Some InDels were detected exclusively by either SAMtools (n = 162,851) or Dindel (n = 245,005). One source of difference between these non-consensus sets was the size of the InDels. SAMtools was able to detect larger InDels and the maximum length detected was 81 bp, whereas the largest InDel detected by Dindel was 56 bp. Another difference was that many variants detected exclusively by Dindel had low quality scores, possibly originating from calling variants associated with poor base or map qualities for which no minimum thresholds were applied as explained above. Moreover, some InDels detected exclusively by SAMtools had low quality, and also low depth of coverage.

### Stringent filtration of consensus InDels defines a high quality set

In order to improve the confidence in the detected InDels, only the consensus set from the two packages were chosen for further filtration and analyses. Among all the variants detected by the two packages, about 74 % (n = 1,110,149) were called by both methods and hence were considered consensus. About 11 % of the SAMtools detected variants and 16 % of the Dindel variants failed to be within the consensus set and were excluded from further analyses.

Even after obtaining the consensus variants, further filtrations were applied to extract a catalogue of high quality InDels. Major filtration criteria were: InDel quality ≥30, coverage ≥5 and ≤ mean coverage in a line + 3 SD, non-reference allele supported by both forward and reverse strands, and gap between consecutive InDels >1 base. This resulted in the retention of 883,411 variants (about 80 % of the consensus set) including 397,438 insertions, 476,793 deletions and 9,180 block substitutions (i.e. substitution of a stretch of consecutive nucleotides with a new block). The number of consensus filtered (CF) InDels varied widely across the chicken lines (Fig. 1; Additional file 1) with the minimum number detected from the inbred line, I5 (n = 171,680) and the maximum number from the white egg layer line, WEL6 (n = 391,796). The average number of InDels detected from brown egg layer (BEL) lines however, was higher (410 K ± 28 K), compared to those from WELs (395 K ±55 K) and inbred lines (357 K ±26 K). This InDel diversity was found to be highly correlated (r = 0.78, *P* < 0.001) with SNP diversity from these lines presented in a recent paper by Gheyas *et al*. [35].

A large proportion of our CF InDels were fixed within lines for the non-reference alleles (allele frequency ≥0.9) and the proportion of these InDel varied widely across the chicken lines ranging from 43 to 87 %. In general, the inbred lines showed the greatest proportion of fixed variants (on average about 82 %) followed by WEL (average 62 %) and BEL lines (average 47 %). The greater proportion of fixed variants in the inbred lines was expected as these lines have been developed by many generations of sib-mating for experimental purposes.

**Fig. 2:**
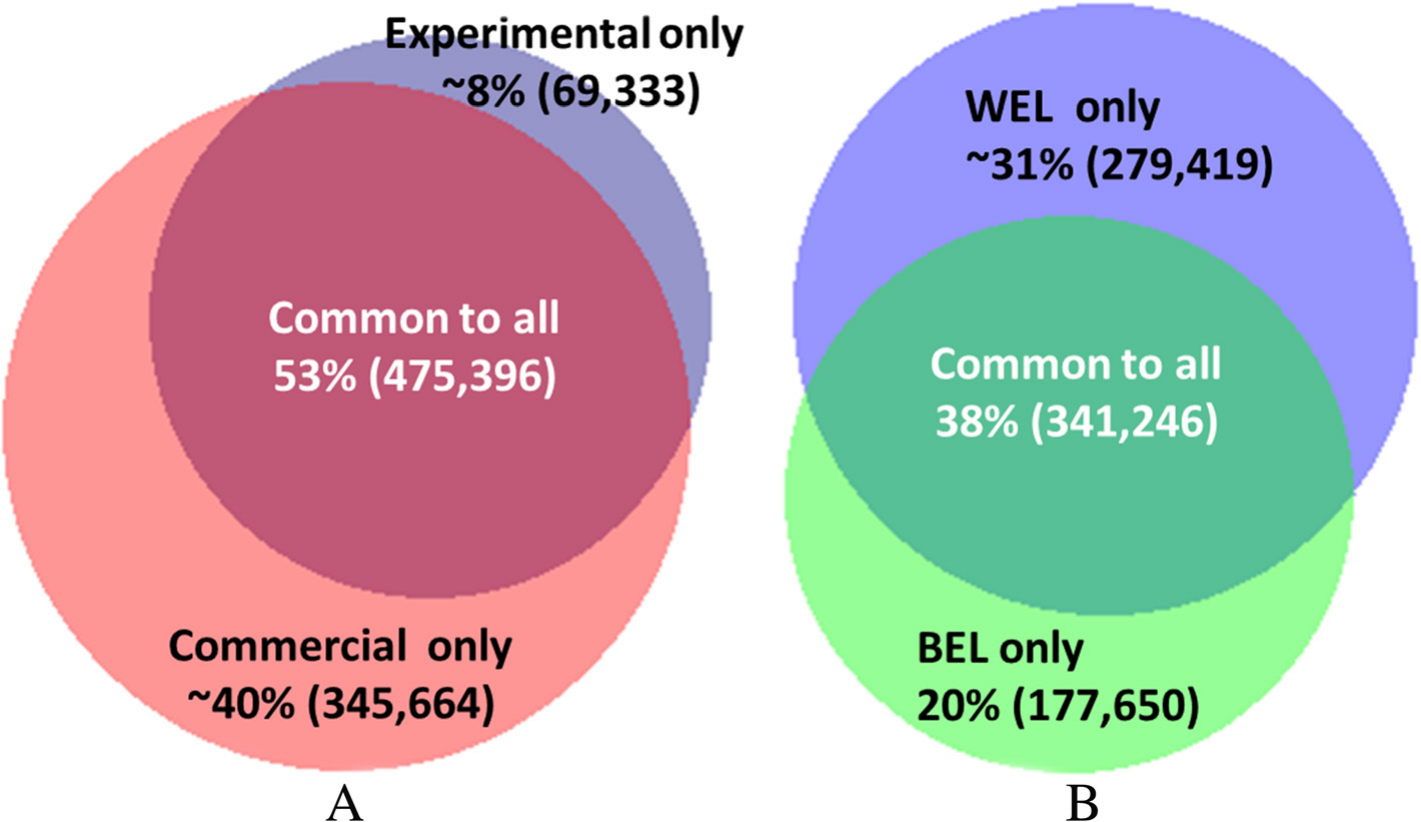
Venn diagrams of InDels shared between (**a**) commercial and experimental lines; (**b**) WEL and BEL. The size of the circles reflects the relative number of InDels detected from each group within the consensus filtered set (n = 890,393)

In Fig. 2, we explored the proportion of variants shared by different groups of chickens, viz., between commercial (consisting of WEL and BEL) and experimental lines (including inbred and RI-J), and between WEL and BEL groups. Each circle of the Venn diagrams represents the percentage of InDels in each of the groups in relation to the CF set. Figure 2a shows that 53 % of the variants were shared between the commercial and experimental lines, while 40 % of the InDels were detected exclusively within commercial lines and 8 % within inbred lines. Similarly Fig. 2b reveals that 38 % of the variants were shared between WEL and BEL groups and about 11 % more InDels were detected from WEL lines compared to BELs.

**Fig. 3:**
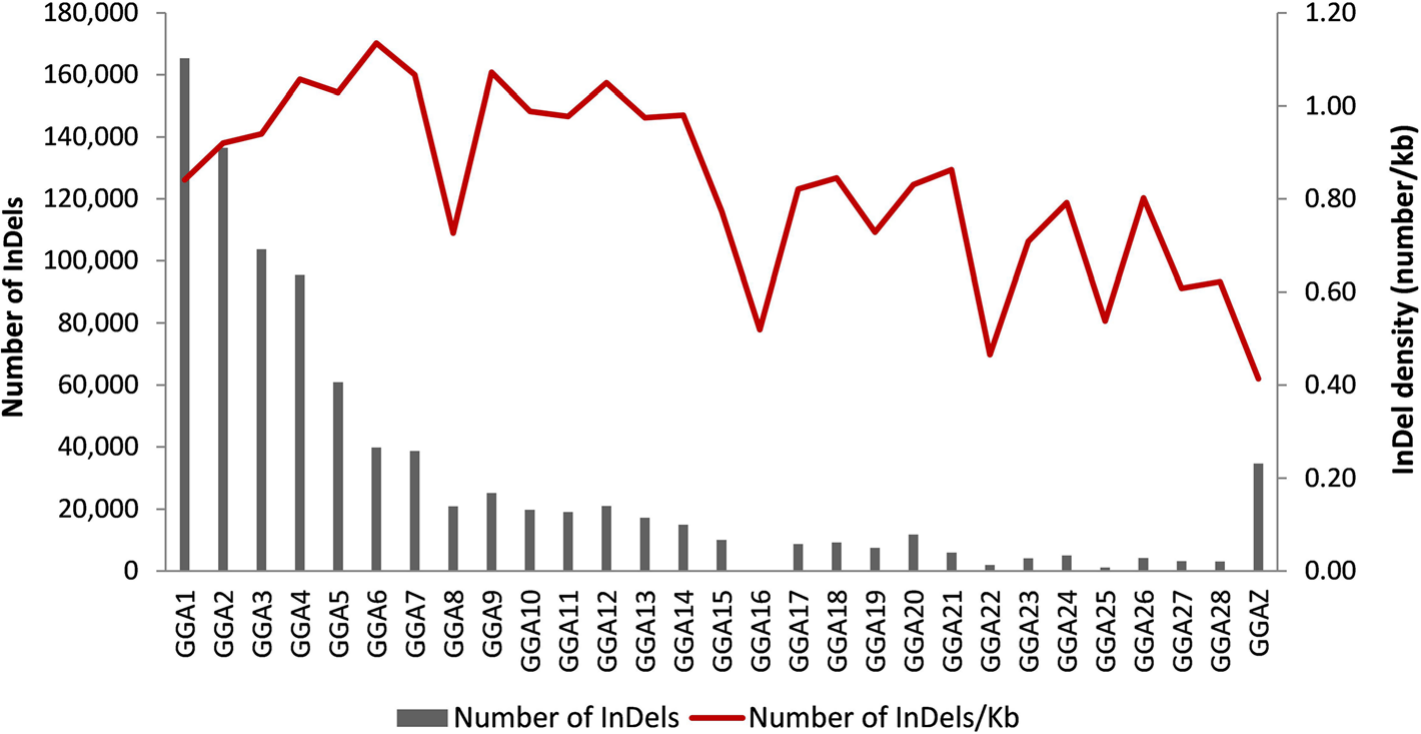
Chromosome-wise InDel counts and densities for the consensus filtered set detected from 16 layer lines. InDel counts are shown with bars and densities (number per kb) using line graph

The densities of InDels varied widely across the chromosomes with a mean of 0.78 (±0.25) InDels per kb (Fig. 3). The lowest density was observed on GGAZ (0.41 per kb) and the highest on GGA6 (1.13 per kb). The microchromosomes showed significantly lower InDel density (*P < 0.*05) than the macro- (GGA1-5) and intermediate chromosomes (GGA6-10).

### InDel validation and false discovery rates

In order to estimate the validation rate of InDels detection in our study, we sequenced 24 randomly selected genomic regions by Sanger method on all the individuals from one chicken line (see Materials and Methods). The total length of sequence data from these regions was 9,760 bp and the analysis of this data generated 22 InDels with good sequence quality and reliable alignment. These InDels were compared with those detected from NGS data from the same regions. Three sets of InDels were compared against Sanger list: (1) the set detected by Dindel; (2) the set identified by SAMtools; and (3) the CF InDel set. This allowed us to simultaneously compare the two InDel calling packages, estimate the validation rates of the InDels detected in the present study, and also to get an estimate of the false negative (FN) rate, i.e. the proportion of true InDels that could not be detected. Results in Table 1 show that SAMtools performed better than Dindel in terms of both the validation and FN rates, even though the latter has been developed specifically for calling short InDels. The validation rates were: 88 % with Dindel, 94 % with SAMtools, and 93 % with the CF set. Similarly, the corresponding FN rates were: 21 % for Dindel, 15 % for SAMtools and 26 % for CF set.

**Table 1:** False discovery rates (FDR) for the InDels identified by next generation sequencing in 24 validation regions

InDel Set	NGS InDels	TP^a^	FP^b^	TN^c^	FN^d^	Sensitivity	Specificity	FP rate (1-specificity)	FN rate (1-sensitivity)	VR^e^
Dindel	17	15	2	9,739	4	0.7895	0.9998	0.0002	0.2105	0.8824
SAMtools	17	16	1	9,740	3	0.8421	0.9999	0.0001	0.1579	0.9411
Consensus filtered^6^	15	14	1	9,740	5	0.7368	0.9999	0.0001	0.2631	0.9333

^a^TP = True Positive and this refers to the number of InDels detected by both Sanger and NGS methods. ^b^FP = False Positive and this refers to the number of InDels detected only by NGS. ^c^TN = True Negatives and refers to the number of bases which were sequenced but not called as InDels by Sanger or NGS. ^d^FN = False Negative and refers to the number of InDels detected only by Sanger. ^e^VR = Validation Rate was calculated as (TP/number of NGS InDels). ^f^Consensus filtered set included the common InDels detected by both SAMtools and Dindel and retained after filtration

### Physical properties of consensus filtered InDels

**Fig. 4:**
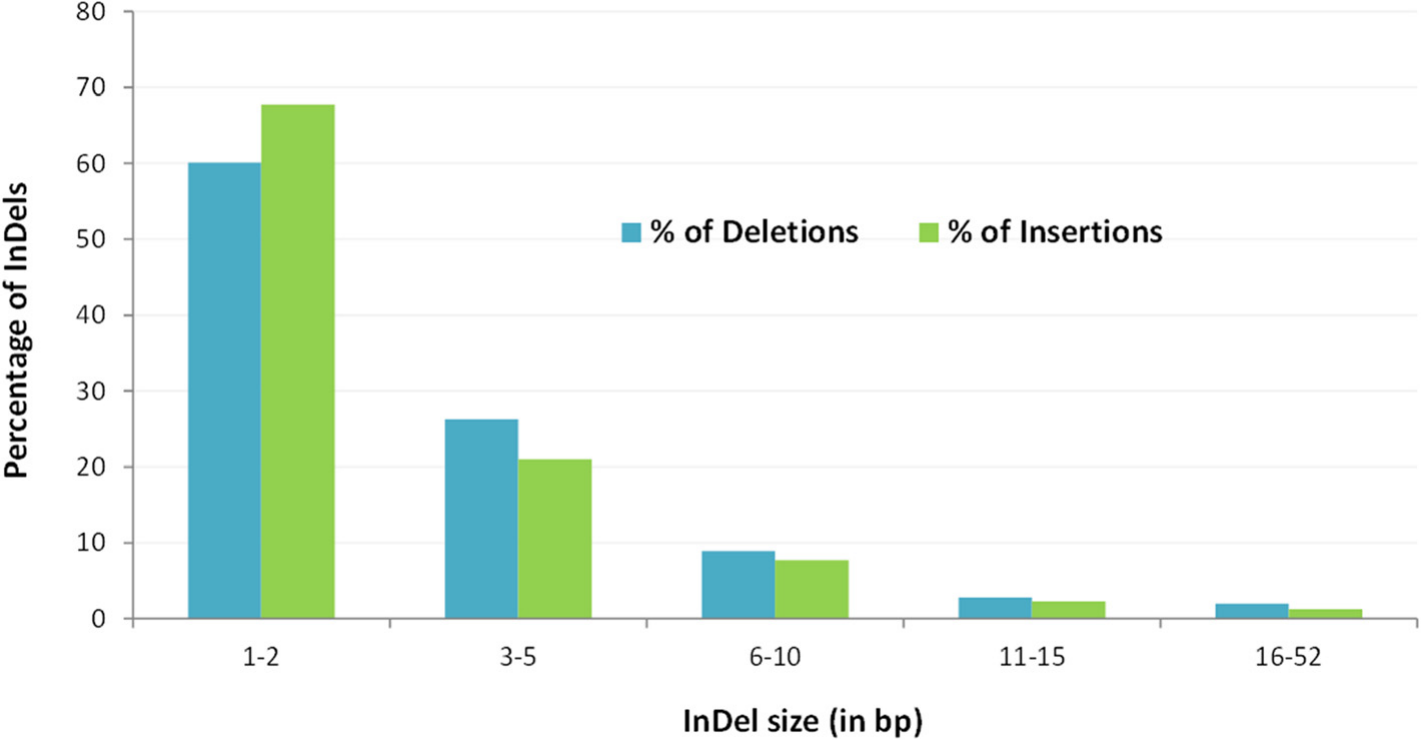
Size distribution of unique insertions and deletions in the consensus filtered set. The percentages were calculated based on total number of unique insertions or deletions

The size of the InDel detectable depends on the length of the sequenced reads. More than 86 % of the insertions and deletions in our study were small with lengths of 1–5 nucleotides and only 1–2 % of variants were above 15 nucleotides (Fig. 4). Over 47 % of the insertions and 38 % of the deletions were the result of only single nucleotide changes. The largest insertion and deletion in the CF set was 33 and 52 nucleotides, respectively.

Apart from length, we also classified the insertions (n = 397,438) and deletions (n = 476,793) based on whether they consisted of non-repeat or repeat expansions. About 65 % of the insertions and 61 % of the deletions were non-repeat in nature consisting of single (47 % insertions and 38 % deletions), two (11 % insertions and 14 % deletions), three (3 % insertions and 5 % deletions) or four or more nucleotides (3 % of both the insertions and deletions). The rest of the InDels consisted of either perfect or imperfect repeat motifs. Those with perfect repeats included: monomeric (14 % for insertions and 12 % for deletions), dimeric (0.5 % for both insertions and deletions) and trimeric motifs (0.2 % for insertions and 0.1 % for deletions). About 21 % of the insertions and 27 % of the deletions consisted of imperfect repeats consisting of combinations of multiple motifs of different order. We did not remove the InDels within tandem repeat sequences as they constituted a substantial proportion.

### Functional annotation of InDels and their predicted biological effects

Annotation of genetic variants against functional elements in the genome (e.g. genes, or non-coding functional elements) is a major step towards their characterisation. In the following sub-sections we describe the annotation of the CF InDels against known genes, predict the effects of coding variants, explore the distribution pattern of the coding InDels in relation of polypeptide chains and also annotate the InDels against most conserved elements (MCEs).

#### InDels within coding and non-coding regions

In order to investigate their possible biological effects, if any, the CF InDels were annotated against the Ensembl chicken gene database (release 71) (see Materials and Methods). This revealed that ~48 % of InDels fell within genic regions and were annotated as intronic (46 %), UTR (1.4 %), exonic (0.12 %), splicing (0.02 %) or ncRNA (0.01 %) variants. The remainder of the InDels were located outside the genic regions and constituted the intergenic (~50 %) and 1 kb up- or downstream (2.7 %) categories (Table 2). In total 1,088 coding variants were detected, which consisted of 613 frameshift (FS), 465 non-frameshift (NFS) and 10 stop-gain/loss InDels. Although the number of FS InDels was higher than the NFS mutations, their ratio (1.3) was found to be much lower compared to that of non-triplet (variants that are not multiple of three nucleotides) and triplet InDels in the non-coding region of the genome (ratio 4.7). We observed that about 54 % of the genes harboring NFS InDels and 56 % of the genes with FS and stopgain/loss InDels had one or more paralogs in the genome. These paralogous genes can, at least partly, compensate for any harmful effect of a protein due to the presence of InDels. It was also observed that 34 % of the FS and stopgain/loss InDels and about 18 % of the NFS InDels were harboured by novel chicken genes for which the functions are still unknown. It is possible that these genes represent artefacts of *Ab Initio* gene prediction method. The much greater proportion of FS and stopgain/loss InDels in novel genes suggests that many of these genes may be prediction artefacts.

**Table 2:** Summary of annotation of consensus filtered InDels based on Ensembl gene annotations (release 71)

	Count	Percent (%)
*Total number of InDels*	*883,570*	
*Annotation possible*	*886,116*	100
*Alternate annotation*	*2,546*	0.29
Annotation result		
Intergenic	438,714	49.51
Intronic	409,956	46.26
Exonic	1,088	0.12
frameshift deletion	*270*	*0.03*
frameshift insertion	*333*	*0.04*
frameshift substitution	*10*	*>0.01*
non-frameshift deletion	*301*	*0.03*
non-frameshift insertion	*162*	*0.02*
non-frameshift substitution	*2*	*>0.01*
stop-gain/stop-loss	*10*	*>0.01*
1 kb downstream	13,854	1.56
1 kb upstream	9,959	1.12
UTR3	11,488	1.30
UTR5	819	0.09
Splicing	162	0.02
Non-coding RNA (ncRNA)	85^a^	0.01

^a^Includes 9 ncRNA variants that were detected by annotation against novel ncRNA transcripts [71]

#### Effects of non-frameshift InDels on protein function

**Fig. 5:**
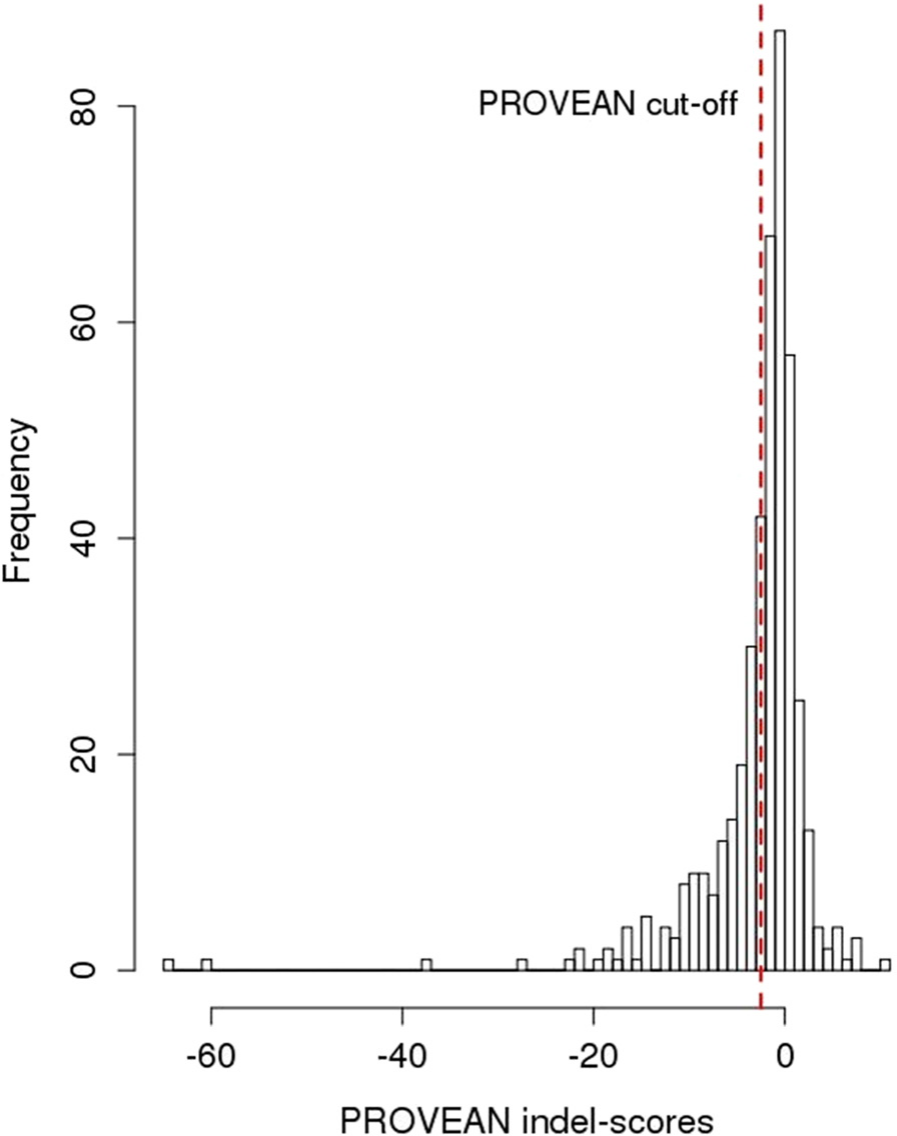
Distribution of PROVEAN scores for non-frameshift InDels. The score of −2.5 was used as the threshold below which an InDel was considered to be intolerant

Unlike the FS InDels, the NFS mutations do not destroy the reading frame of the protein but only insert or delete one or more amino-acid(s). As a result, the NFS mutations can still be sustained without major effects on protein function if the affected amino acid is non-essential for biological activity. In order to investigate if the NFS mutations are likely to have implications on protein function or not, we used the PROVEAN [46] software to predict the potential effect of NFS-InDels (see Materials and Methods). The PROVEAN method calculates a score for each NFS InDel based on the degree of change in the alignment score of homologous proteins due to the introduction of the variant in question. Although these scores are considered to be correlated with the fitness (in evolutionary terms) of the variants, a default score of −2.282 was used as a cut-off point below which any InDels were predicted “Intolerant” in evolutionary terms. Figure 5 shows the distribution of the PROVEAN scores for the NFS InDels. Using the default threshold, about 29 % (n = 153) of the NFS variants were predicted to be evolutionary intolerant. A small proportion (~1.5 %) of the InDels exhibited extreme low scores (below −20) indicating that these may have more disruptive effects than the others.

#### Relative positions of coding InDels within polypeptide chains

**Fig. 6:**
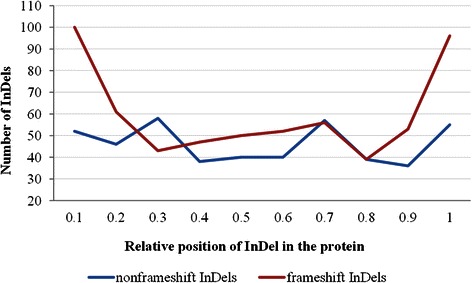
Relative location of frameshift and non-frameshift InDels in polypeptides. The relative position was calculated by dividing the position of an InDel within a polypeptide with the length of the polypeptide

The biological effect of a coding InDel is expected to be a function of its relative position within polypeptide chain. InDels located at the N- and C-termini may have less biological effects. In the case of a C-terminal InDel, most of the protein product would be translated before encountering the variant while in the instance of a N-terminal InDel, there may be an opportunity to rescue major part of the protein by using a cryptic downstream start codon [47]. Investigation of the relative positions of the coding InDels revealed that the FS variants occurred more frequently either at the N-terminal (n = 100 out of 619 FS InDels) or C- terminal parts (n = 96) while the NFS mutations showed a more or less uniform distribution across the length of the polypeptides (Fig. 6). Even, most of the NFS InDels with extreme PROVEAN scores (> −20) were generally located in the middle of proteins rather than at the ends. Only one of the seven such extremely low-scored NFS InDels was found to be located at the beginning of the polypeptide sequence. We investigated high frequency FS InDels (n = 72 with frequency >0.9), which were located at the N-terminal part of the protein (i.e. located <0.1 relative protein length) and noticed that about 96 % had another downstream ATG start codon located very close to the InDel site (Additional file 2). These start codons may help to rescue the major part of the proteins by acting as a cryptic translation initiation site.

#### InDels within conserved elements in the genome

**Fig. 7:**
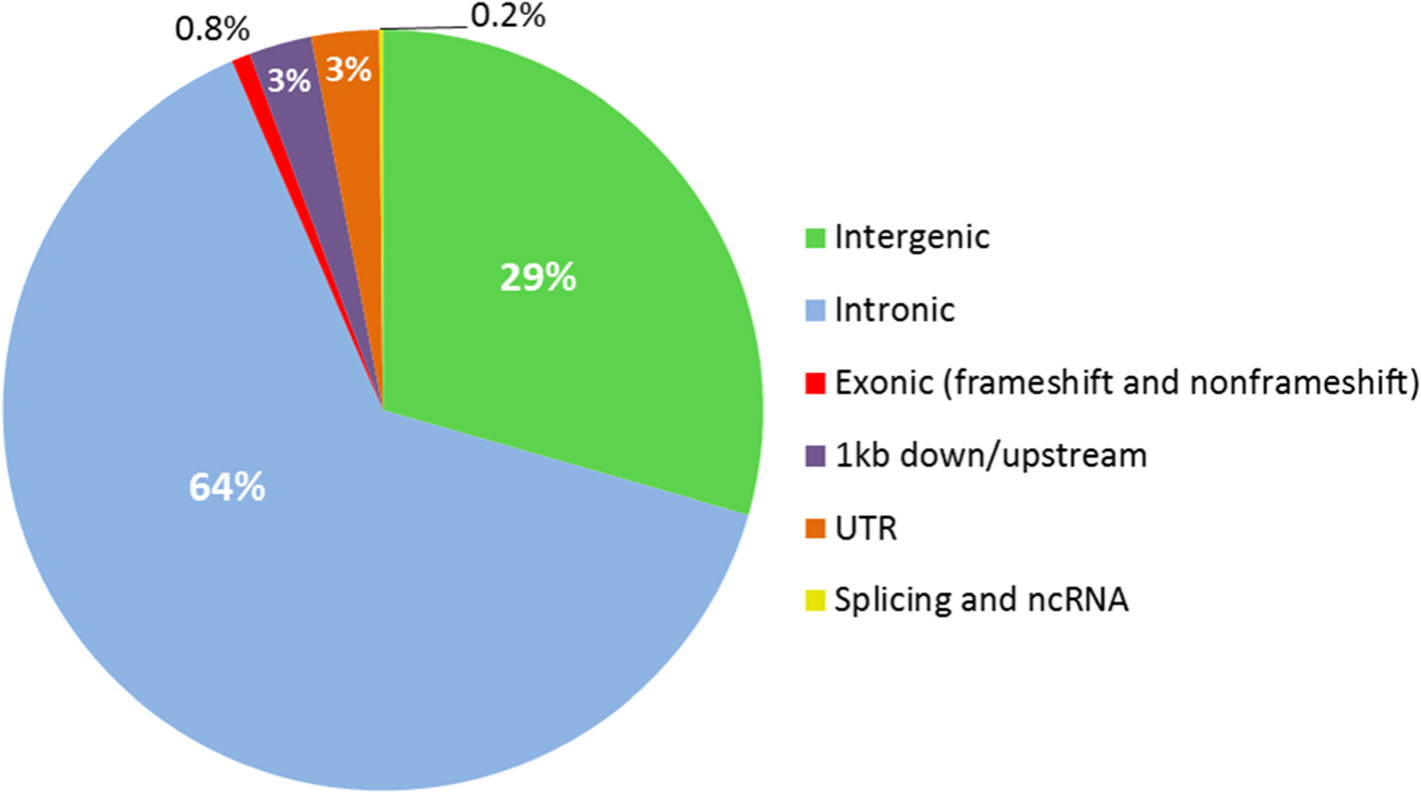
Categorisation of the InDels within most-conserved-elements (MCEs)

Apart from the coding variants, mutations within non-coding regions may have a biological effect if they coincide with promoters, enhancers or other functional regulatory elements. The chicken genome, however, is still poorly annotated for non-coding functional elements and as a result it is difficult at this stage to characterize the variants within these regions and predict their possible biological effects. In the absence of comprehensive annotation of genomes, researchers have searched for evolutionary conserved regions as a surrogate to detecting regions potentially under purifying selection and hence are likely to be functional [48]. In our study we annotated the CF InDels against the list of MCEs in the chicken genome [49]. These MCEs, downloaded from UCSC database, were identified from multiple alignment of the genomes of six distantly related species namely human, mouse, rat, opossum, *Xenopus tropicalis* and zebrafish. The dataset contain 950,084 MCEs between 1 and 4,280 nucleotides in length and covering 68 Mb of the chicken genome. About 2.5 % of the CF InDels (n = 22,671) overlapped with MCEs. The majority of InDels in MCEs belonged to intronic regions (64 %) followed by intergenic (29 %), UTRs (3 %), up- or downstream regions (3 %), and only a small proportion (0.8 %) from exonic regions (Fig. 7). The MCE-intronic variants may represent uncharacterized exons or affect the regulation of transcriptional activity or splicing efficiency of their host genes [50]. Similarly, variants within UTRs and up- or downstream regions may also have regulatory effects on gene expression. The density of InDels in MCEs (0.34 per kb) was much lower compared to the overall mean density in the genome (0.78 per kb) confirming the expected purifying selection acting on these conserved elements.

### Allele frequency pattern of different InDel categories

**Fig. 8:**
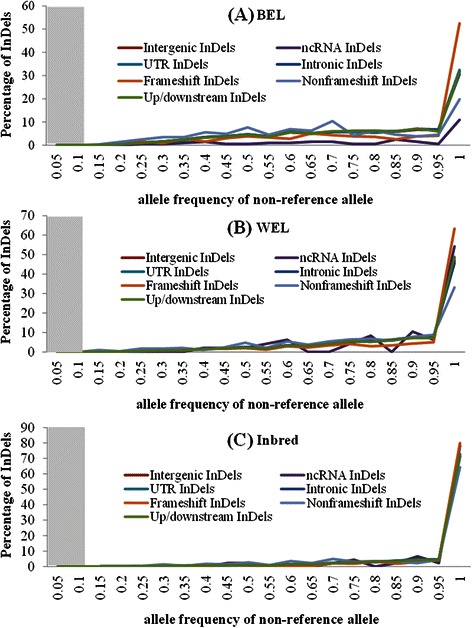
Frequency distributions of non-reference (alternative) alleles of different InDel categories from three chicken groups. The InDel categories included were: intergenic, intronic, ncRNA, UTR, up/downstream, frameshift and non-frameshift. (**a**) BEL = brown egg layer, (**b**) WEL = white egg layer and (**c**) Inbred. The shaded region denotes the AAF range without any InDel detected (<1 %)

**Fig. 9:**
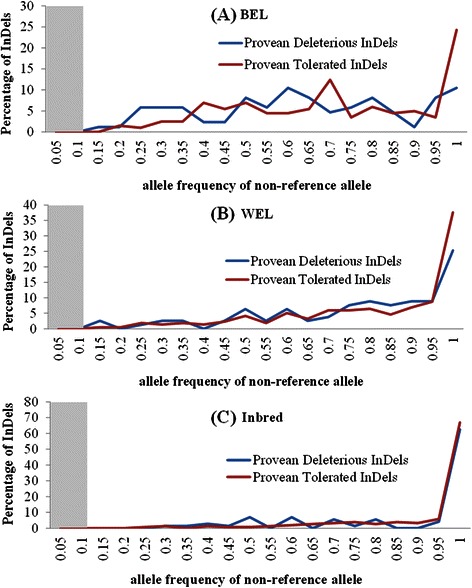
Frequency distributions of non-reference (alternative) alleles of intolerant and tolerant InDels from three chicken groups. The intolerant and tolerant InDels were predicted by the PROVEAN method. (**a**) BEL = brown egg layer, (**b**) WEL = white egg layer and (**c**) Inbred. The shaded region denotes the AAF range without any InDel detected (<1 %)

**Fig. 10:**
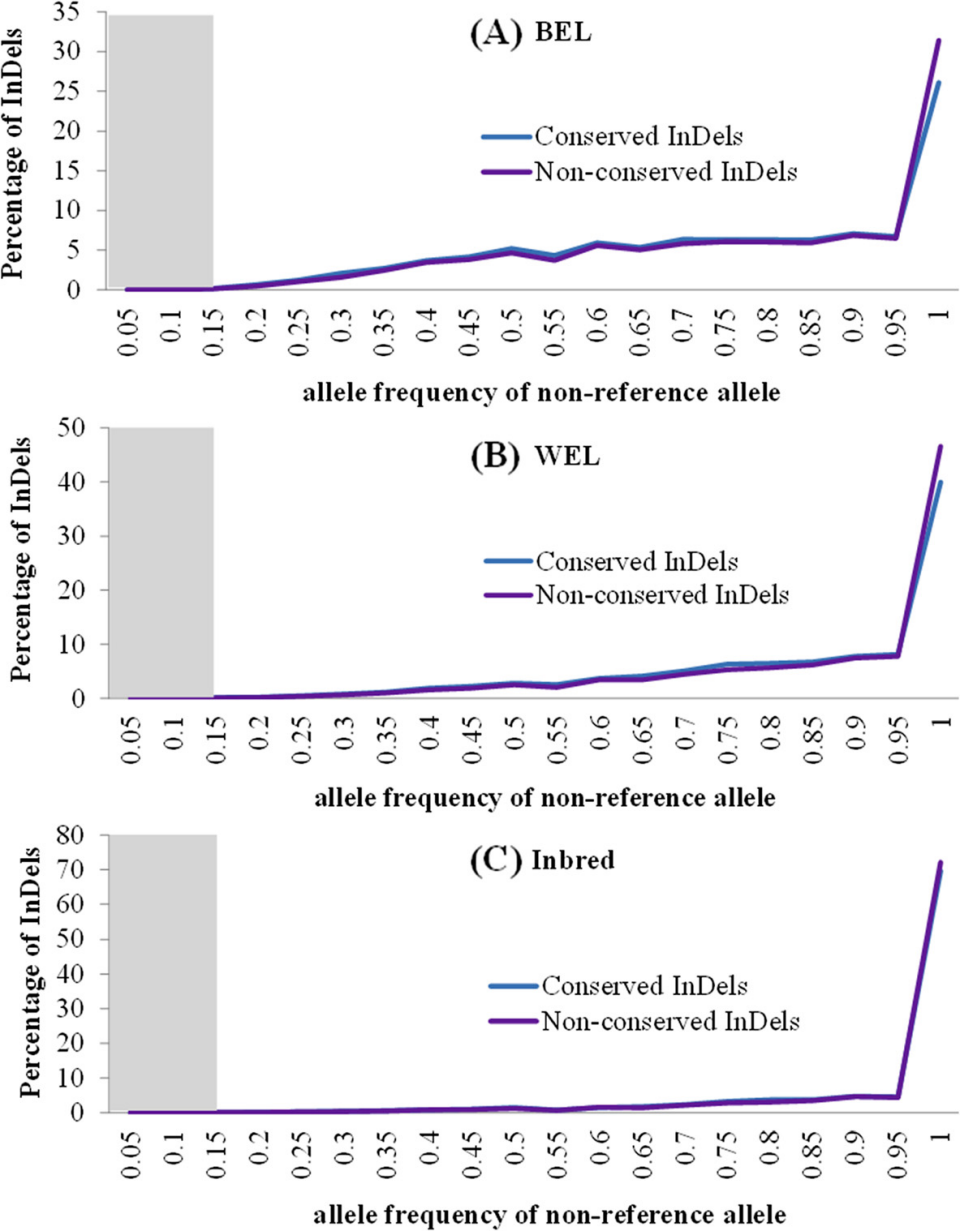
Frequency distributions of non-reference (alternative) alleles of conserved and non-conserved InDels from three chicken groups. (**a**) BEL = brown egg layer, (**b**) WEL = white egg layer and (**c**) Inbred. The shaded region denotes the AAF range without any InDel detected (<1.5 %)

Estimating the allele frequency of variants is an important step towards their characterization. The extent of phenotypic impact that a functional variant can exert at population level depends on its frequency. We, therefore, explored the frequency distributions of non-reference or alternative alleles (AAF) from different InDel categories in the three chicken groups, viz. WEL, BEL and inbred (Figs. 8, 9, 10). These figures emphasize two major points: (i) there are almost no variants in the lower frequency range (AAF < 0.1) and (ii) in all chicken groups, irrespective of the InDel categories - whether potentially functional or neutral - the distributions are right-skewed indicating that most of the detected InDels are present in high frequency. The fixation (AAF ≥ 0.9) of a large proportion of variants, however, is more pronounced within the inbred group as expected compared to the commercial chickens (Fig. 8).

In the Additional file 3 we present a heatmap using the mean AAF of putative functional InDels (viz. the variants categorized as exonic, splicing, ncRNA and those coinciding with MCEs) that are fixed (AAF ≥ 0.9) in at least one of the chicken groups. Out of 14,033 InDels used for creating the heatmap, about 94 % overlapped with one or more QTLs specified in chicken QTLdb (http://www.animalgenome.org/cgi-bin/QTLdb/GG/index). For instance, about 80 % of these InDels coincided with egg-related traits (e.g. egg production, shell quality, age and weight at first egg, etc.), 56 % coincided with bone-related traits (e.g. mineral content, strength, weight, etc.), 36 % overlapped with antibody response to various diseases and 25 % overlapped with feed conversion ratio or feed efficiency traits. About 16 % of the InDels presented in the heatmap were fixed in all of the three groups and most probably represented old variants. On the other hand, there were variants that were fixed in only a specific group while segregating at much lower frequency (e.g. ≤0.5) or not detected at all in other groups; their proportion varied with 7.3 % in BEL, 10.3 % in WEL and 8.4 % in inbred lines. These group-specific variants may be associated with traits relevant to that particular chicken group.

### Enrichment of functional classes of genes with potential loss-of-function mutations

In order to investigate if the potentially loss-of-function (LOF) mutations (viz. the FS, stop-gain/loss, and intolerant-NFS InDels) are enriched within certain groups of genes with functional similarity, we performed a clustered analysis of the genes using the DAVID Gene Functional Classification tool [51]. Performing the analyses separately on the three chicken groups showed enrichment of different classes of genes. We selected only those classes with enrichment scores (ES) ≥1.3 and genes with kappa score ≥0.75 (as suggested by DAVID) that indicate strong agreement between genes. This resulted in the retention of one class from each group. The class from the WEL group was related to cell proliferation, chromosome and Golgi organization, and epithelial cell adhesion. The gene class from the BEL group was related to cell differentiation, spermatogenesis, muscle contraction and blood coagulation and the class from the inbred group was related to cell proliferation and respiration. These classes consisted of 12 genes in total (Additional file 4). Five of these genes were shared by at least two groups: the gene *STK31* was common to all three groups; the genes - *SMCHD1, TBRG4* and *DDX31* were common between the inbred and the WEL groups; and the gene, *DCLK3* was shared by the WEL and BEL groups. The rest of the genes were present exclusively in one particular group: the genes *AQR* and *KIFC3* in the WEL group, and the genes *MAK*, *PBK*, *SRMS*, *PRKCH* and *DES* in the BEL group*.*

We observed that one FS InDel within significant gene classes was identified from multiple lines and was present at very high frequency. This variant (insertion of a single T at the position GGA2:46501278) was located within the first exon of the gene *DCLK3* (*doublecortin gene family*) and was detected from all the eleven lines from the WEL and BEL groups and reached near fixation, indicating possible selective advantage for these chicken groups. On the other hand, three InDels from the significant gene classes were detected at very high frequency (AAF = 1) only within single lines with the possibility to have functional implications within those specific lines. These were: a FS insertion of a single C (at the position GGA11:483395) in the exon 9 of the *KIFC3* gene from a single WEL line, an intolerant NFS deletion at the position GGA5: 31527889 from the *AQR* (or *IBP160*) gene from another WEL line, and a FS insertion of a single C (at the position GGA7:21670698) in the exon 7 of the *DES* (*desmin*) gene from a BEL line.

## Discussion

This study aimed to detect and characterise high quality InDels from the chicken genome by screening multiple commercial and experimental layer chicken lines. A major strength of our study was the use of multiple birds (10–15) from each of the populations analysed, which allowed better characterisation of the detected variants e.g. provided the opportunity to explore the allele-frequency patterns of InDels. Although in a recent study Yan *et al*. [38] reported about 1.3 M InDels, they only used single bird from each of the 12 breeds analysed and did not investigate to any depth the functional characteristics of the InDels. For instance, they made no attempt to predict the functional effects, if any, of non-frameshift InDels, or to estimate allele frequency patterns or to find InDels overlapping with conserved elements. Our study provides a more comprehensive functional characterization of the InDels using most of the currently available resources and genomic databases on chicken. This study is also the first detailed InDel characterization in any avian species, and hence can serve as an important resource for other birds.

Since InDel calling inherently suffers from multiple problems, we adopted a number of approaches to ensure a high degree of fidelity in our calls including taking only the consensus variants detected by two different callers, and applying a number of stringent filtration criteria. This approach was highly successful as we observed a very high rate of validation (93 %). This validation rate is better than that reported in the similar recent study (88 %) by Yan *et al*. [38]. The number of InDels used for validation in our study, however, was small and as a result, the rates may vary on larger dataset.

Some of the shortfalls in our study originated from the use of a pooled-sequencing approach. Although the major strength of pooled sequencing is that it facilitates the screening of many individuals within limited budget and time, it has certain drawbacks. One major drawback is that this approach is prone to miss rare and low frequency variants when sequencing is performed at low coverage [52, 53]. This was observed in our study too by the large estimate of false negative rate (26 %) and by the absence of rare and low frequency variants in allele frequency graphs. The sequence coverage in our study was low- only 7-17X for pooled samples of 10–15 individuals per line. This coverage however, dropped further during downstream analyses when only good quality reads were recruited for variant calling. This factor was further compounded with the use of stringent filtration criteria, especially, the use of at least five reads covering an InDel site and the support of non-reference allele by at least one read from both the strands. Apart from these factors, it is generally more difficult to detect heterozygous InDels compared to homozygous ones when the sequencing coverage is low [54–56]. According to an estimate based on single sample sequencing, it would require coverage of at least 20X to detect 99 % of the heterozygous variants [55]. These details explain why in our study we have seen mostly high frequency InDels in all groups of chickens and in all annotation categories. However, we concede that the allele frequency estimation from pooled sequencing may not be accurate as it is impossible to assess if all the samples had equal contribution in the reads covering InDel sites. Besides the frequency estimates are likely to be upwardly biased due to use of only 10–15 individuals per line for the estimations. In spite of all these issues, we argue that we were able to shed some light on the frequency spectrum of the InDels and the findings suggest that we have detected mostly common variants. We would also like to emphasize that it is the common variants that are most likely to have major implications for poultry breeding.

The main findings observed about our CF InDels were: (1) majority were small in size (1–5 nucleotides), (2) a substantial proportion was located within tandem repeats, (3) large proportions were fixed within lines, (4) the InDel densities varied widely across chromosomes, (5) higher number of frameshift than non-frameshift variants were detected but their ratio was much lower compared to that of non-triplet and triplet InDels in the non-coding regions and (6) FS mutations were located more frequently either near the N- or C-terminal part of the proteins compared with NFS mutations. Most of these observations are consistent with the findings of other studies. For instance, previous studies on chicken have also found majority of InDels to be within small size range of 1–10 nucleotides [33, 36, 38]. Similarly, several studies in humans and chickens have reported excess of InDels to be present within tandem duplicates [20, 36]. The proportion of fixed InDels (43 to 87 %) within lines is a function of level of inbreeding, although the large rate of fixation observed in our study is most probably inflated due to the use of limited number of individuals and also due to the difficulty in detecting heterozygous InDels as discussed above. During our InDel call we removed those InDels which were fixed in all or most of the lines (see Materials and Methods) to ensure that these are not errors in reference genome.

The present study revealed that micro-chromosomes had significantly lower density of InDels compared with macro- and intermediate sized chromosomes. This is contrary to the expectation based on the higher recombination rates in microchromosomes [57, 58] as studies on other species have revealed that recombination rate is positively correlated with polymorphism rate [59, 60]. When we compared the InDel densities with the SNP densities in chromosomes - using the SNPs described by Kranis *et al*. [34]- we observed a different picture; the micro- and intermediate chromosomes had significantly higher (*P < 0.*05) densities of SNPs compared to that in macrochromosomes. This indicates that even though SNP density is affected by recombination rates of the chromosomes, for InDel density other factors, such as selection pressure probably play a more important role. It is likely that the high gene content in the microchromosomes promotes purifying selection against harmful mutations like InDels [57, 58]. Chromosome 16 is an example of rich harbour of many important genes or gene clusters viz. the major histocompatibility complex (MHC), nucleolus organiser region (NOR), olfactory receptor (OR), cysteine-rich domain scavenger receptor (SRCR) and putative immunoglobulin-like receptors [61]. This might have prompted the purifying selection to keep the InDel rate in this chromosome as one of the lowest (0.52 per kb) among the autosomes. Besides, this chromosome is rich in duplicated regions, which possibly have affected the alignment of sequence reads leading to detection of very few InDels in the first place [57]. Chromosome Z also showed a very low density of InDels. In contrast to the microchromosomes, the low density of InDels in GGAZ is a reflection of reduced overall genetic diversity observed in this chromosome due to a number of possible reasons such as selection on sex-linked characters and low male effective population size [62, 34]. Similar to our findings, several other studies have also reported lower densities of InDels in micro- and sex chromosomes [36, 38]. Besides, the sequenced birds consisted of both male and female samples and as a result the depth of coverage for sex chromosomes is expected to be less than that of autosomes, which may also had an impact on the number variants detected from these chromosomes.

The detection of relatively higher number of FS compared to NFS InDels is consistent with the results from previous studies on different species [38, 63]. Non-triplet InDels (such as FS) are at least twice more likely to be generated by chance alone compared to the triplet InDels (e.g. NFS). However, when present in coding-regions, non-triplet InDels can be highly disruptive as they cause complete change in the sequence of C-terminal part of the protein or may uncover a stop codon to truncate the translated product. The peptides produced by FS mutations have been found to be associated with many diseases [64–67]. As a consequence, a much lower proportion of non-triplet variant is expected in coding regions due to purifying selection compared to that in non-coding regions. Our finding conformed to this expectation as the ratio of non-triplet and triplet InDels was about 4 times less in coding regions compared with that in non-coding parts.

The observation that FS mutations appeared more frequently at the beginning or end of the proteins corroborates with the understanding that FS mutations in the middle of the proteins will have more harmful impacts than those at the end. However, one caveat of this finding is that the presence of many FS InDels at the extreme ends of proteins may reflect wrong annotation of genes and their corresponding translation start site in the current gene database. It is possible that these InDels are actually located outside the boundary of coding region. To assess this possibility, we investigated some randomly selected genes (ca. 10) with FS mutations that were located at the beginning of the protein (position ≤0.1 relative to polypeptide length). Blastp analyses of the proteins encoded by these genes showed that in most cases these are known proteins in other species. However, when we predicted the translation start sites of these genes with the NetStart 1.0 programme [68], in most cases (9 out of 10 genes checked) different initiation codons were predicted from the ones specified in Ensembl. In all these cases, the FS InDels were located before the NetStart predicted translation initiation site, indicating the possibility of inaccurate gene annotation. On the other hand, when we randomly checked 10 genes with NFS mutation as a control, in most cases the NetStart predicted initiation sites were same as those given by Ensembl. However, correcting the annotation of coding regions is beyond the scope of the present study and would require further work, such as a genome-wide proteomic investigation.

In the present study we identified a number of gene classes that were significantly enriched with potentially harmful mutations and some of the FS and intolerant-NFS InDels within these gene classes were present in high frequency in multiple or single lines. It is difficult to explain how genes associated with important physiological functions can harbour such harmful mutations. However, presence of paralogs for majority (67 %) of these genes can be a possible explanation as these can help to compensate for the loss-of-function caused by harmful mutations. We explored if the fixed FS and NFS-InDels overlapped with any chicken QTLs as this might help find association of these variants with important traits. We found that the FS insertion (GGA2:46501278) in the *DCLK3* gene that was near-fixed in both WEL and BEL groups*,* coincided with QTLs associated with a wide range of traits such as tibia bone mineral content, egg production, blood quality, fatness, etc. as specified in Chicken QTLdb (http://www.animalgenome.org/cgi-bin/QTLdb/GG/index). The FS insertion (GGA11:483395) detected from the *KIFC3* gene from an individual WEL line overlapped with QTLs associated with tibia area and stress, intestine length and carcass traits. The intolerant NFS mutation (GGA5: 31527889) detected in the *AQR* gene from a WEL line and the FS insertion (GGA7:21670698) from the *DES* gene overlapped with QTLs associated with fatness traits, egg production, carcass traits, antibody responses to diseases, etc. Overlap with QTLs associated with many traits suggests possible pleiotropic effects of these genes and their functional variants. An alternative possibility to selective advantage is that these InDels are moderately (or lowly) harmful under the environmental conditions where the birds are maintained but have attained fixation through hitchhiking by being linked to some beneficial mutations under strong positive selection [69].

The CF InDels detected in our study were compared with those available in the public domain (dbSNP build 140) and those recently described by Yan *et al.* [38]. When the comparison was made based on coordinates alone, about 81 % of our CF variants were found to be shared with the already known InDels. However, when the allelic information was combined with the coordinates for comparison, the percentage of shared variants reduced to about 53 %, indicating that the non-reference allele at an InDel site may differ among populations. The major reason for the lower sharing of alleles between studies is that a substantial proportion of these InDels are tandem repeats that are prone to be highly variable. For instance, about 59 % of these non-shared variants due to allele were perfect tandem repeats of monomeric nature (i.e. repeats of either A, T, C or G), while many others were imperfect or complex repeats. Another possible reason can be the misalignment of the reads, particularly when majority of these inconsistent alleles consisted of tandem repeats. To investigate this possibility, we randomly checked about 50 InDels and most of them appeared true InDels with correct alignments and good coverage in our study. The observation of large proportion of common InDels across studies has served as a validation that most of these InDels are true variants.

## Conclusions

This study provides a large catalogue of small insertion and deletion genetic variants and their detailed characterization by analysing many chickens from diverse commercial and experimental layer lines. Use of consensus InDels from two different bioinformatics packages followed by stringent filtration criteria provided confidence in the detected set and the FDR estimation suggested a high rate of validation. Moreover, overlap of a large proportion (~81 %) of the 883 K InDels detected in the present study with Yan *et al.* [36] is a further proof that majority of the InDels detected in our study and those present in the public domain are correct. This paper adds about 168 K novel InDels over what has already been detected from chicken genome by previous studies. Most importantly, this study provides an in-depth characterisation of the InDels by adopting different approaches and using available resources. To the best of our knowledge, this is the first study on chicken to shed any light on the frequency spectrum of detected InDels and it indicates that majority of the InDels detected so far from chickens by different studies are probably high frequency, common variants. The results of this study also suggest that sequencing at much higher coverage will be required to detect rare and low frequency InDels.

The resource created in this study is expected to have major implications in future studies not only in chicken but also in other avian species. For instance, the large catalogue of InDels along with their functional characteristics can help gain insights into the genetic variant profile of chicken genome and in the identification of causal variants underlying various diseases and other important traits. Particularly, potentially functional variants that have been found to be fixed or near fixed in different groups can be further explored to understand their possible effects on phenotypes.

## Methods

### Whole genome re-sequencing of layer chickens lines

The NGS sequence data used for InDel detection was generated under a previous study [34]. In brief the data was generated by Illumina sequencing of 163 chickens originating from 11 commercial and 5 experimental layer lines (Additional file 1). The samples from commercial lines were supplied by Hy-line International and Lohmann in the Synbreed Consortium and consisted of 6 lines of white egg layers (WEL1-6) and 5 lines of brown egg layers (BEL1-5). Other samples originated from four inbred lines (Wellcome, N, 15 and 0 lines) from the Institute of Animal Health (now The National Avian Research Facility (NARF), Edinburgh) and an unselected brown leghorn line from the Roslin Institute (RI-J line). For each line, except WEL6, 10–15 individuals were sequenced as pooled DNA using a paired end protocol. For WEL6, however, three samples were sequenced individually. The length of paired reads varied from 76–101 nucleotides. Sequencing was performed with a mean observed coverage of 8-17X per line. Further details on library preparation, sequencing and alignment can be retrieved from Kranis *et al.* [34]. We mapped detected InDels on the published chicken reference genome (Gallus_gallus_4.0).

### InDel calling by SAMtools

The InDel calling with SAMtools (version 0.1.18) [39] was performed using its *mpileup* function and BCFtools. The *mpileup* function computes the likelihood of the data given each possible genotype and stores the likelihoods in BCF format (binary variant call). BCFtools then applies the prior and does the actual variant calling. We used the following commands for calling the variants using SAMtools.

samtools mpileup **–**q20 **–**Q20 **-**AB **-**ugf <referenceFile.fa> <bamFile.bam> | bcftools view **-**bvcg><var.raw.bcf>

bcftools view <var.raw.bcf> | vcfutils.pl varFilter **-**D99999> <var.flt.vcf>

In these commands, the –q and –Q options were given to specify the minimum thresholds for base and map qualities to be 20. The option **–**A was used to force the analysis of all the reads including anomalous read pairs to avoid issues in the analyses such as sudden stops. The option **–**B was used to disable the BAQ (Base Alignment Quality) calculation as it is computationally very demanding. Disabling BAQ may result in increased quality scores in some false positive SNP calls close to InDels, but it will not affect the alignment process in InDel calling. The maximum depth of coverage was limited to 99999 by the option –D.

### InDel calling by Dindel

InDel calling using the Dindel package (version 1.01) [18] consisted of four stages:

#### Stage 1– extraction of all candidate InDels

dindel --analysis getCIGARInDels –bamFile < bamFile.bam > --outputFile < outFile-stage1 > --ref < referenceFile.fa>

Extra step in Stage 1– reduction of the number of candidate InDels:

python selectCandidates.py -i <outFile-stage1> −o <outFile-extrastage>

#### Stage 2– grouping the candidate InDels into windows of ~120 bp

python makeWindows.py --inputVarFile <outFile_extrastage> --windowFilePrefix <outFile-stage2> --numWindowsPerFile 20000

#### Stage 3– generation for every window of candidate haplotypes from candidate InDels

dindel --analysis indels --doPooled –bamFile <bamFile.bam> --ref <referenceFile.fa> --varFile <outFile-stage2> --libFile <librarieFile> --outputFile <outFile-stage3>

#### Stage 4 – production of a VCF4 file format with the InDels calls:

python mergeOutputPooled.py --inputFiles <outFile-stage3> --outputFile <outFile-stage4> --ref <referenceFile.fa> --numSamples 3–15 --numBamFiles 1

We used this procedure for calling InDels from a pool, and the default parameters which included the selection of candidate InDels seen at least twice (in the stage to reduce the number of candidate InDels).

### Filtration of consensus InDels

We obtained the common or consensus set of InDels from the two InDel callers, SAMtools and Dindel, by comparing the co-ordinates of the InDels from these two packages. The following filtration criteria were then applied on the consensus set to reduce the number of false-positives: (i) InDel quality score ≥30 based on SAMtools provided score; (ii) coverage at the InDel position ≥5 and ≤ mean coverage in a lines + 3 SD (standard deviation); (iii) the non-reference allele supported by both forward and reverse strands; and (iv) gap between consecutive InDels >1 base. Finally, we removed those InDels which are fixed for the non-reference allele in all or most of the lines (14–16 lines) as these may actually represent possible sequencing errors in reference genome or alignment error. Most of the consensus filtered InDels (n = 865,597) have been submitted to dbSNP (NCBI) using the handle “DWBURT” with the submitter batch ID “Chicken_indel_dwburt” (http://www.ncbi.nlm.nih.gov/projects/SNP/snp_viewBatch.cgi?sbid=1062064).

The large InDels (>50 bp) and the majority of block substitution InDels have been submitted to EVA database (provisional accession number PRJEB9374; http://www.ebi.ac.uk/eva/).

### Calculation of FDRs for InDels

We estimated the FDRs of InDel calling by resequencing several random regions with the Sanger method followed by comparing the detected InDels from the Sanger and NGS data from these regions. For Sanger sequencing, we initially selected 28 regions from different chromosomes (GGA1-6, 10–12, 15–17, 19, 21, 22, 24, 26, 28 and linkage group LGE22C19W28_E50C23). All the ten chickens from the RI-J line were individually sequenced using the Sanger method. The sequences were mapped to reference genome using the long read mapping algorithm in BWA and also separately with DNASTAR MegAlign™ (http://www.dnastar.com/t-megalign.aspx) programme with default parameters. The BWA alignment was used with SAMtools to obtain the InDel locations fast, whereas the trace files from MegAlign^TM^ were used to manually check the correctness of alignments and for manual calling of the variants.

Low confidence Sanger InDels i.e. those with poor sequence quality, poor alignment or lacking support from both forward and reverse strands were excluded from the calculations. Three regions from GGA10, 15 and 17 had to be removed due to missing or poor quality sequence from one or more samples by Sanger method even after repeated attempts. Further, one region from GGA3 was removed due to alignment issue, leaving 24 regions for estimation of FDRs.

Based on the comparison of Sanger and NGS variants from the same region an InDel was called true positive (TP) when it was detected by both NGS and Sanger, a false positive (FP) when it was detected only by NGS, a false negative (FN) when it was detected only by Sanger. Any sequenced bases which were not called as InDels by either Sanger or NGS were considered to be true negatives (TN). Based on these, FDR calculations were defined as follows: Sensitivity (rate of true InDels correctly identified by NGS) = TP/(TP + FN); Specificity (rate of true non-InDels that were correctly recognized by NGS) = TN/(TN + FP); False positive rate (FPR) = (1-specificity); and False negative rate (FNR) = (1-sensitivity). The proportion of false positive InDels in the filtered NGS list was calculated as (total number of NGS InDels – total number of InDels detected by both NGS and Sanger)/total number of NGS InDels).

### Functional annotation and effect prediction of InDels

The genomic positions of the InDels and their effect on protein coding regions were predicted by annotating them against the Ensembl gene annotation database (release 71) for chicken. The software ANNOVAR (version July 06, 2012) [70] was used for this purpose. In addition, we also annotated the InDels against 1,608 novel non-coding RNA transcripts (ncRNA), which have recently been characterized [71]. To identify if any of the InDels coincided with these ncRNA transcripts we used the BEDtools (version 2.17.0, http://bedtools.readthedocs.org/en/latest/).

The InDels within the coding regions (non-frameshift and stop-gain/loss InDels) were further analysed using the Protein Variation Effect Analyser (PROVEAN, version 1.1) [46], which predicts whether an amino acid substitution, insertion or deletion are likely to affect protein function or not. PROVEAN was run with the default parameters on the NCBI non-redundant protein database. Delta score of −2.5 was used as the threshold below which any InDel was predicted “Deleterious” or “Intolerant” in evolutionary term.

The filtered list of InDels were also annotated using ANNOVAR against the PhastCons predicted “Most Conserved Elements” (MCE) for chicken. For this annotation, however, we first had to map the InDels against the previous version of chicken reference sequence (Gallus_gallus_2.1) as the MCE dataset contains co-ordinates in relation to this reference build. It was possible to map unambiguously only 585,154 (66 %) InDels on Gallus_gallus_2.1 and as a result we could only annotate these variants. The MCE data were downloaded from UCSC database: ftp://hgdownload.cse.ucsc.edu/goldenPath/galGal3/database/phastConsElements7way.txt.gz. This data contains PhastCons scores [48] for chicken (Gallus_gallus_2.1 or 3, May 2006).

The DAVID Gene Functional Classification tool [51] was used to find if any particular classes of genes were enriched for frameshift and intolerant non-frameshift mutations in the BEL, WEL and inbred chicken groups. For this analysis, we selected only those genes with kappa score ≥0.75 and clusters with enrichment scores ≥1.3.

### Venn diagram, heatmap and allele frequency estimation

Venn diagrams of InDels shared between different lines were created by the BioVenn tool (available at http://www.cmbi.ru.nl/cdd/biovenn/). Estimation of allele frequency of InDels was based on the proportion of high quality reads supporting the non-reference alleles. Mean frequencies within groups (e.g. BEL, WEL and Inbred) were calculated based on the populations where the InDels were detected. Heatmap of fixed InDels (AAF ≥ 0.9) was generated by the programme, Genesis [72].

## Additional files

Additional file 1:
**An excel file with the details of the chicken lines analysed for InDel detection and the number of InDels detected from each line using the SAMtools and Dindel programmes and after getting the consensus set.**

Additional file 2:
**Scatterplot showing the positions of high frequency (AAF ≥ 0.9) frameshift InDels (in X axis) located at beginning of cDNA and of the nearest downstream ATG start codon (in Y axis).** Both the start codon and InDel positions are represented as relative to the cDNA lengths. Data from 72 FS InDels which were located within 0.1 length of cDNA have been used to create the graph.
Additional file 3:
**A figure with a heatmap of fixed InDels in at least one group (BEL, WEL or I) for different categories (FS, NFS, stop-gain/loss, splicing, ncRNA and MCE) by Genesis tool.**

Additional file 4:
**An excel file with the details about the gene clusters enriched with frameshift and PROVEAN predicted intolerant non-frameshift InDels from three chicken groups viz. white egg layer (WEL), brown egg layer (BEL) and Inbred.** The DAVID Gene Functional Classification tool was used to identify these gene clusters.
