# Supplementary material for: Detection and characterization of small insertion and deletion genetic variants in modern layer chicken genomes
Source: BMC Genomics. 2015 Jul 31;16:562. doi: 10.1186/s12864-015-1711-1 (PMC4563830; doi:10.1186/s12864-015-1711-1)

Additional file 2: Scatterplot showing the positions of high frequency (AAF≥ 0.9) frameshift InDels (in X axis) located at beginning of cDNA and of the nearest downstream ATG start codon (in Y axis). Both the start codon and InDel positions are represented as relative to the cDNA lengths. Data from 72 FS InDels which were located within 0.1 length of cDNA have been used to create the graph.

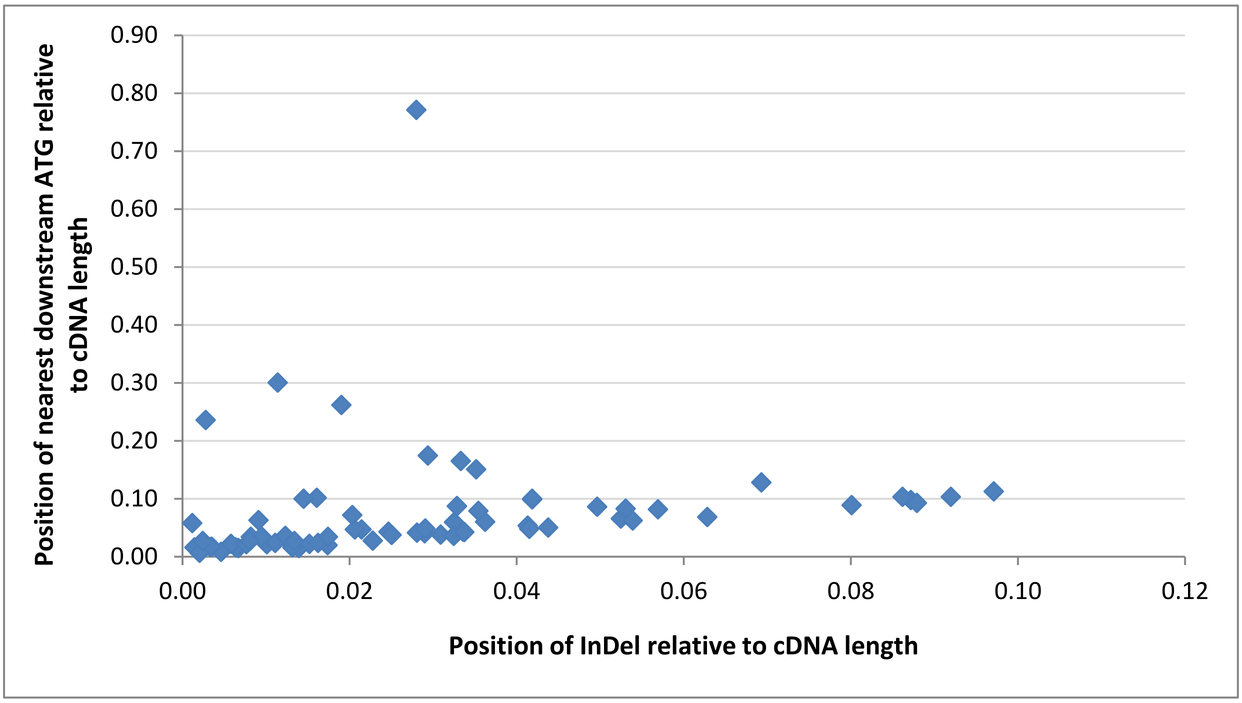

Supplement: Additional file 2: — Scatterplot showing the positions of high frequency (AAF ≥ 0.9) frameshift InDels (in X axis) located at beginning of cDNA and of the nearest downstream ATG start codon (in Y axis). Both the start codon and InDel positions are represented as relative to the cDNA lengths. Data from 72 FS InDels which were located within 0.1 length of cDNA have been used to create the graph. [file 12864_2015_1711_MOESM2_ESM.pdf]
